# Demographic Differences and Trends of Vitamin D Levels Among the Teenaged Girls in Balochistan

**DOI:** 10.7759/cureus.12335

**Published:** 2020-12-28

**Authors:** Gulalai Rehman, Hajra Ahmad, Allau Ddin, Habibullah Rind, Seemin Kashif, Amna Saleem, Saliha Kakar, Muhammad Samsoor Zarak

**Affiliations:** 1 Nutrition, Balochistan Institute of Nephrology and Urology Quetta, Quetta, PAK; 2 Environmental Design, Health, and Nutritional Sciences, Allama Iqbal Open University, Islamabad, PAK; 3 Surgery, Bolan Medical College, Quetta, PAK; 4 Nephrology, Balochistan Institute of Nephrology and Urology Quetta, Quetta, PAK; 5 Nutrition, Allama Iqbal Open University, Islamabad, PAK; 6 Internal Medicine, Bolan Medical College, Quetta, PAK; 7 Epidemiology and Public Health, Bolan Medical College, Quetta, PAK

**Keywords:** vitamin d deficiency, pakistan, balochistan, adolescent, vitamin d

## Abstract

Background

Vitamin D is a vital micronutrient and plays a vital role in defining the bone mineral density of an individual. There are many factors that regulate the levels of vitamin D in our body. The deficiency in vitamin D leads to various complications, with the most important one weakening of bones. Adolescence defines the degree of bone mineral density, which reduces with the growing age in a gradual fashion.

Methods

The study was a cross-sectional study conducted in Zarghoon town, Quetta, Pakistan. A sample size of 142 was taken from urban and rural areas. Participants were adolescent girls falling in the age bracket of 13-18 years. The circulating level of 25-hydroxy vitamin D was assessed using the ELISA (enzyme-linked immunosorbent assay) technique. Data were analyzed with SPSS Version 20 (IBM Corp.).

Results

Overall, vitamin D deficiency was 32.4%, and 9.9 % of girls were found to be severely deficient, where the highest proportion belonged to urban samples. The prevalence rate of vitamin D insufficiency was 39.4%. The urban population had a higher prevalence of low levels of vitamin D. In urban respondents, 49.1% had an insufficient vitamin D level, 33.3% had a deficient vitamin D level, and 17.5% had a severely deficient vitamin D level. In rural respondents, 47.1% had normal vitamin D levels, 32.9% had insufficient vitamin D levels, 15.3% were deficient, and 4.7% were severely deficient.

Conclusion

It was concluded that vitamin D deficiency has a high prevalence among adolescent girls of school age. Additionally, it is more prevalent in urban areas than in rural areas.

## Introduction

Vitamin D is a vital micronutrient of our body. In the last few years, there is an amplified consideration of vitamin D because of the emergence of vitamin D deficiency leading to osteomalacia and rickets, a major issue of health worldwide. In addition, many researchers proved that vitamin D has a role not only in the skeletal system but also in the extraskeletal system. There is promising evidence that vitamin D has a role in the prevention of many cancers [1]. Vitamin D deficiency leads to various chronic diseases such as cardiovascular disease, diabetes mellitus [2], hypertension, and chronic kidney diseases [3]. Present-day research has compelling evidence that vitamin D has a very important role in the prevention and treatment of autoimmune diseases [4].

Adolescence is an incredibly important phase for bone mineral expansion and is thought a primary window for the avoidance of long-term consequences of low bone mineral content. Likewise, bone mineral density in adolescent girls affects postmenopausal osteoporosis. Physical activity is the primary modifiable stimulus for increased bone growth as it decreases calcium excretion and increases calcium absorption [5].

More than 50 billion teenagers are either deficient or insufficient in vitamin D in America and more than 50% of children are vitamin deficient living in Europe, Saudi, India, and China [6]. Prevalence of vitamin D deficiency rated high in apparently healthy adolescent girls due to less sun exposure, use of sunscreens, and nutritional deficiency due to decreased milk intake and increased consumption of soft drinks and juices [7]. Though there are some data to support a role for confounding from sun exposure, D levels are associated with obesity, and the main factors could be less sun exposure, lack of outdoor activities [8], and deposition of vitamin D in adipose tissue [9].

The prevalence of vitamin D deficiency in adolescents is high in many countries, especially Southeast Asia [10]. There is growing evidence that vitamin D deficiency is increasing in Pakistan, as evidenced by the Pakistan National Nutritional Survey in 2011 that reported a deficiency in 83% women and 41% children.

Adolescence is a very important age for calcium absorption and bone accretion. The upsurge of calcium and bone during this period depends on the intake of vitamin D [7]. There are very few studies available that are carried out on adolescents. Balochistan is even worse, as not a single study is available despite the fact that growth stunting reported by the national nutritional survey is highest in the territory. Therefore, the authors aim to assess the levels of vitamin D among the adolescent girls of Balochistan. In order to provide an extended window, we intended to work in two settings, i.e., rural and urban population to provide more understanding on the frequencies of deficiency of vitamin D.

## Materials and methods

This is a cross-sectional study that included a sample of 142. The study was conducted in Quetta, the capital of Balochistan province of Pakistan. Quetta is the capital of the province of Baluchistan. Quetta is divided into two towns, i.e., Zarghoon and Chiltan. The study locale was secondary schools in urban and rural areas of Zarghoon town Quetta. The target population was adolescent girls aged 13-18 years. The consent was taken from the parents of the participants. The participants' blood sample was collected only once, and the vitamin D status was found out using the ELISA (enzyme-linked immunosorbent assay) method. The ethical approval was sought from the Bolan University of Medical and Health Sciences and Department of Health, Balochistan.

The duration of the study was six months (March 10, 2019, to September 15, 2019). The sampling was done using multi-stage simple random sampling. In the first step, the list of all the schools situated in urban and rural areas of the Zarghoon town was obtained, and through balloting three schools from the urban area and three schools from rural areas were selected. In the second step, a list of all the girls' names was taken from the attendance register, and the sample was chosen randomly from that list. A total of 150 girls were selected, out of which eight were dropped due to their reasons. Hence, the total sample of the completers was 142. The statistical analysis was performed using SPSS Version 20 (IBM Corp., Armonk, NY, USA).

The vitamin D level was assessed through testing the circulating blood levels of 25-hydroxy vitamin D. A phlebotomist acquired the blood sample. ELISA was used to detect the level of vitamin D in the blood. A vitamin D level of >30 ng/mL is considered normal. The cut-off for insufficient vitamin D level is 20-29 ng/mL; however, levels < 20 ng/mL indicate vitamin D deficiency [11].

## Results

The total number of samples was 142, of which 57 were from the urban areas and 85 were from rural areas. The representation of participants was 59.9% from the rural area and 40.1% from the urban areas. In the rural population, the age groups of 15 and 16 years had a comparatively higher proportion, whereas in the urban population, the age group of 14 years had maximum representation. The rural sample's mean age was 15.9 years, whereas in an urban area, it was 15.4 years (SD: ±1.3). In the entire sample, the mean age was 15.7 years (SD: ±1.3) (Table 1).

**Table 1:** Demographics of the sample

Age (years)	Rural %	Urban%	Total %
14	18.8	36.8	26.1
15	22.4	14.0	19.0
16	22.4	24.6	23.2
17	21.2	17.5	19.7
18	15.3	7.0	12.0

Table 2 shows the median value for 25-hydroxy vitamin D levels. Urban sample had a mean value of 8.410 (SD: ±4.16), with minimum and maximum being 3.80 and 20.00, respectively. In the rural sample, the mean value was calculated to be 26.161, with minimum and maximum being 12.40 and 51.6 respectively (SD: ±6.8).

**Table 2:** Distribution of vitamin D levels according to the location

Level of vitamin D	n	Mean	Minimum	Maximum	SD
Urban	57	8.4105	3.80	20.00	4.16
Rural	85	26.1612	12.40	51.60	6.83
Total	142	19.036	3.8	51.6	10.53

Table 3 shows the characterization of vitamin D levels among the girls from the urban and rural areas of the study. The girls from rural areas had a higher percentage of normal Vitamin D levels, whereas vitamin D was at insufficient levels among the girls from urban areas of the study. Overall, the prevalence rate of insufficiency was 39.4%. The deficiency of vitamin D was 32.4%, and 9.9% of the girls were found to be severely deficient, where the highest proportion belonged to the urban sample.

**Table 3:** Characterization of vitamin D levels in urban and rural girls

Vitamin D levels	Rural, % (n)	Urban, % (n)	Total, % (n)
Normal: more than 30 ng/mL	47.1 (40)	0 (0)	28.2 (40)
Insufficient: 20 to 29.9 ng/mL	32.9 (28)	49.1 (28)	39.4 (56)
Deficient: less than 20 ng/mL	15.3 (13)	33.3 (19)	22.5 (32)
Severely deficient: less than 12 ng/mL	4.7 (4)	17.6 (10)	9.9 (14)
Total	100 (85)	100 (57)	100 (142)

Table 4 showed that the p-value of Levene’s test is 0.002; therefore, the study concluded that the variance in vitamin D level of adolescent girls in urban and rural settings is significantly different. The mean difference is 17.7507. The positive t-value indicates that the mean vitamin D level of girls from rural areas is significantly greater than the mean vitamin D level of girls from urban settings. We can conclude that there was a significant difference in the means of vitamin D level in urban and rural adolescent girls (t = 19.218).

**Table 4:** Independent samples test

Level of vitamin D	Levene's test for equality of variances		t-test for equality of means						
	Fisher’s exact test	Significant	t-test	Degree of freedom	Significant (two-tailed)	Mean difference	Standard error difference	95% confidence interval of the difference	
								Lower	Upper
Equal variances assumed	10.042	0.002	17.542	140	0.000	17.7507	1.0119	15.7501	19.7512
Equal variances not assumed			19.218	138.897	0.000	17.7507			

## Discussion

An enormous volume of literature reports that the prevalence of vitamin D deficiency is quite high across the nations. Though a lot of studies have been carried out in different nations and ethnicities, data related to the population of Pakistan are limited. Our research is novel and is the first ever in Balochistan exploring vitamin D levels in adolescent girls in the age group of 13-18 years. The study has compared the vitamin D levels in urban and rural settings of these areas of Quetta.

Previous studies showed that vitamin D deficiency was highly prevalent in the urban population of Pakistan [12]. Metropolitan cities of Pakistan like Karachi are reported to have vitamin D deficiency of greater than 80% among females [13,14]. In our study, the overall prevalence rate of vitamin D deficiency was 32.4%, and 9.9% were found to be severely deficient. Moreover, the prevalence rate for insufficiency was 39.4%. The trends in the rural population were different from the urban population. The rural community had a relatively higher proportion of normal vitamin D levels, i.e., 47.1%, whereas the insufficiency of vitamin D was 32% and deficiency was 20%. However, the urban population had vitamin D deficiency of 50.8% and insufficient vitamin D level of 49.1%. Therefore, in our study, 100% of participants had a low vitamin D level in the urban area.

There are different factors attributed to this deficiency, including lack of sunlight exposure due to cultural dress codes and veiling, pigmented skin, and less time spent outdoors, hot weather, and lower vitamin D intake [10]. The difference in the vitamin D level between urban and rural adolescent girls was striking in our study.

Studies conducted in European countries and India showed that the trends of vitamin D deficiency are similar to our findings. The HELENA study by Valtuena et al. assesses the vitamin D levels in the adolescents of nine European countries in the age bracket of 13-17 years and reported vitamin D deficiency to be 42%. At the same time, insufficiency was 39% in their population. In this study, they recruited all the adolescents who were not taking vitamin D supplements [15]. India has a similar prevalence of vitamin D deficiency in urban areas [16,17].

Comparing our results to the developed countries shows a change in the trend of vitamin D deficiency. More than 50 billion teenagers are either deficient or insufficient in vitamin D in America and more than 50% of children are vitamin deficient living in Europe, Saudi, and China. In the Caucasian population, deficiency of vitamin D was 19% and insufficiency was reported to be 36% [17]. The lifestyle of the participants was assessed to be a predictor for vitamin D deficiency. The results showed that body adiposity and vitamin D level are inversely related to each other [9]. The results are contradictory in the Caucasian population due to the usage of supplements and fortification of food.

There are several limitations to our study. The sample of the study is limited; more extensive sample studies may be needed to generalize the results. The sample of the population belonged to middle and low socioeconomic groups where diet and living lifestyle may be contributing factors. Therefore, the results may not be generalized to the economically privileged population.

## Conclusions

This study provided a statistically significant trend in the distribution of vitamin D levels among adolescent girls. The results are substantial and provide baseline data for policymakers. This is the only study carried out among teenaged girls in Baluchistan so far. The study concluded that vitamin D deficiency has a high prevalence among adolescent girls of school age. Additionally, vitamin D deficiency was found more in the urban areas than in the rural areas.
